# Refractory pneumothorax and hemothorax associated with metastatic scalp angiosarcoma

**DOI:** 10.1186/s40792-020-01001-w

**Published:** 2020-10-22

**Authors:** Masahide Isowa, Satona Tanaka, Ryo Nakanobo, Yoshito Yamada, Hiroshi Date

**Affiliations:** Department of Thoracic Surgery, Kyoto University Hospital, Shogoin-kawahara-cho 54, Sakyo-ku, Kyoto, 606-8507 Japan

**Keywords:** Scalp angiosarcoma, Pneumothorax, Hemothorax

## Abstract

**Background:**

Pulmonary metastasis of scalp angiosarcoma (SA) is a rare, but life-threatening disease, challenging to diagnose and manage. We report two cases of pneumothorax and hemothorax with pathologically proven metastasis of SA in the parietal pleura, which was not predictable from images and difficult to manage.

**Patient A:**

A 73-year-old man with SA underwent chemoradiotherapy and surgical resection for primary skin lesion, was sent to our department to treat right empyema, which was developed during chest tube drainage for pneumothorax. Computed tomography (CT) showed multiple bullous lesions. We performed repetitive video-assisted thoracoscopic surgery (VATS) for the debridement and hemostasis; however, hemothorax was uncontrollable. The repeated cytology of pleural effusion showed no malignancy. We eventually performed fenestration and metastatic SA was pathologically diagnosed by the biopsy of parietal pleura. The patient developed respiratory failure and uncontrolled anemia, which were fatal.

**Patient B:**

A 71-year-old man with SA previously treated with chemoradiotherapy was referred to our department for left pneumothorax. CT showed multiple bullous lesions at apex without any changes at parietal pleura. VATS was performed and the apex bullous lesion with air leakage was resected. The parietal pleura showed several dark-red spots and the biopsy was undertaken. The pathological diagnosis was a metastasis of SA along with visceral pleura and parietal pleura. The patient then developed right pneumothorax and left hemopneumothorax. Bilateral pleurodesis was ineffective and the patient died due to deteriorating general condition.

**Conclusions:**

In patients with a history of SA who develop pneumothorax and hemothorax, metastatic SA to visceral and parietal pleura should be always considered. Surgical biopsy, not cytology, is needed for pathological diagnosis. Lesions in the parietal pleura prior to hemothorax were thoracoscopically observed in one case. Surgeons must recognize that conventional surgical intervention or pleurodesis will have unsatisfactory results.

## Background

Scalp angiosarcoma (SA) is a rare and aggressive malignancy of the skin, which sometimes metastasizes to the lung and causes pneumothorax. For thoracic surgeons, it is difficult to diagnose and manage this disease due to its scarcity and unusual presentation on imaging. In addition, hemothorax is another manifestation of metastatic SA; however, the intrathoracic appearance of pleural lesions causing hemothorax has not been reported.

## Case presentation

### Patient A

**Fig. 1:**
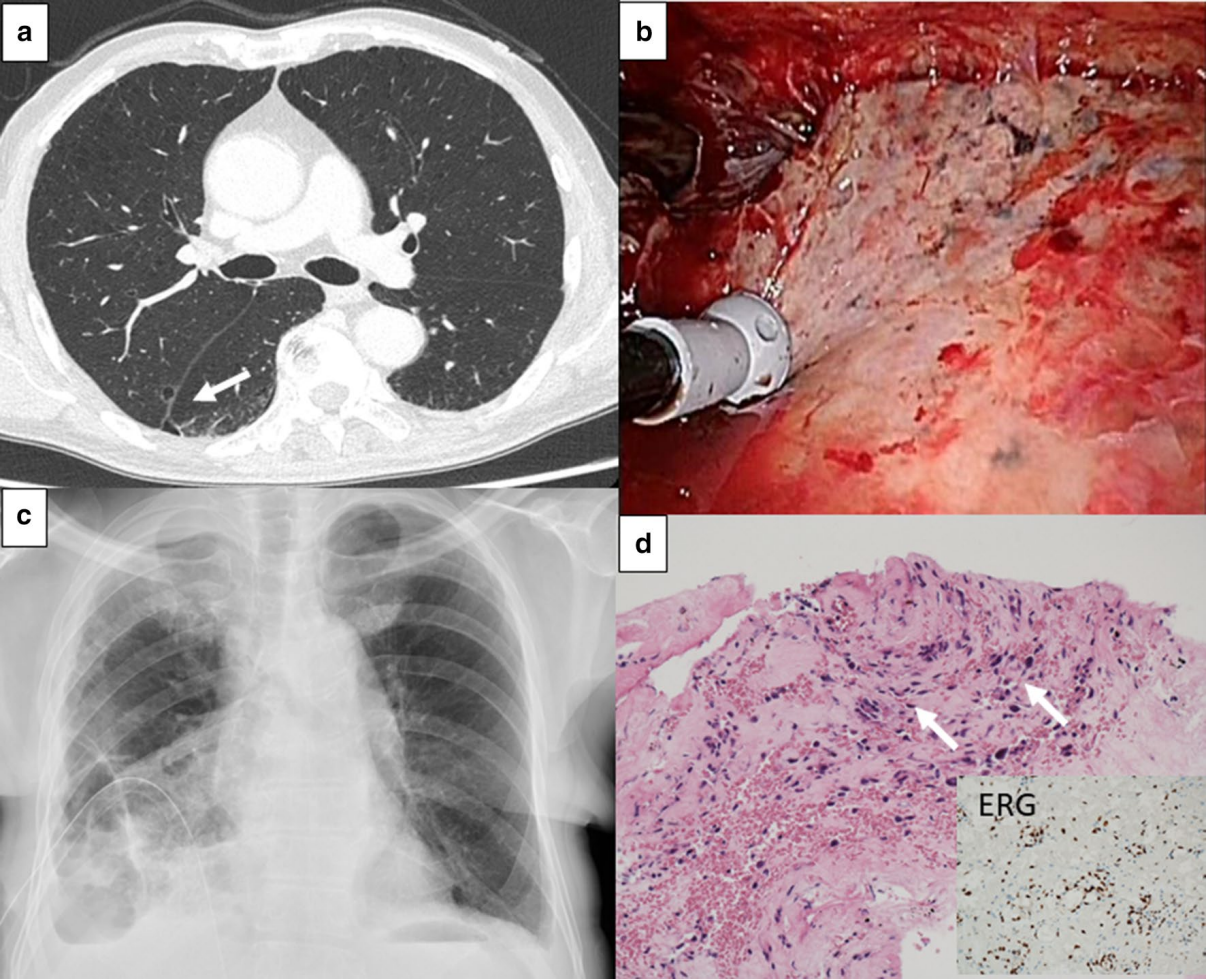
**a** CT, performed 2 months before diagnosed with pneumothorax, showed bullae lesions at the edge of the right upper lobe and right lower lobe (arrow). **b** Intraoperative findings demonstrated fibrinopurulent empyema. **c** Chest X-ray showed expanded right lung; however, dark-red pleural effusion of more than 500 ml per day continued. **d** Pathological findings showed atypical spindle-like cells infiltrating the parietal pleura (arrows). Erythroblast transformation-specific-related gene (ERG) was positive in tumor cells, suggesting the differentiation of endothelial cells

A 73-year-old man with SA underwent chemoradiotherapy (6 courses of weekly Paclitaxel followed by radiation, and 9 courses of monthly Paclitaxel) and surgical resection for primary skin lesion, was sent to our department to treat empyema 11 months after initial diagnosis. Computed tomography (CT) showed multiple bullous lesions (Fig. 1a). We placed a chest tube for right pneumothorax, and the air leakage stopped; however, loculated pleural effusion and methicillin‐resistant Staphylococcus aureus (MRSA) was detected from the subcutaneous abscess around the site of tube insertion, which suggested acute empyema emerged during chest tube drainage. We performed video-assisted thoracoscopic surgery (VATS) for debridement of fibrinopurulent empyema (Fig. 1b). After this surgery, the patient had uncontrollable abundant dark-red pleural effusion (over 500 ml per day; repeated cytology showed no malignancy). We performed re-surgery for the debridement and hemostasis, but the bloody pleural effusion was not controlled (Fig. 1c). Pleural effusion obtained at VATS debridement was positive for MRSA. Finally, since uncontrolled infection was suspected, we decided to perform fenestration. The biopsy of the parietal pleura showed metastatic SA pathologically (Fig. 1d). The patient developed respiratory failure and uncontrolled anemia and died about 2 months after the first surgery.

### Patient B

**Fig. 2:**
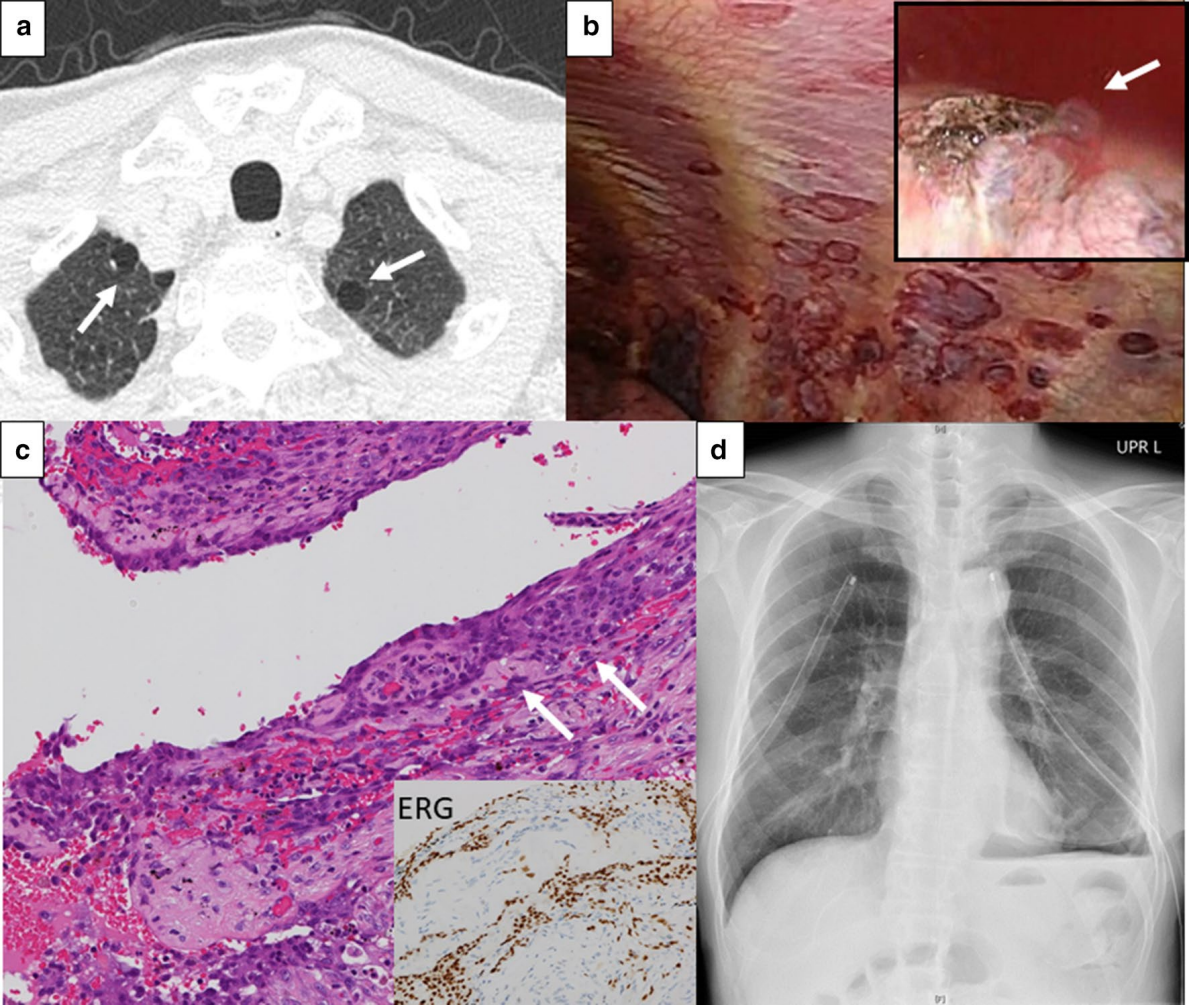
**a** CT, performed 1 week before diagnosed with pneumothorax, showed bullae lesions in the apex (arrows). **b** Intraoperative findings demonstrated bulla in the left apex (arrow) and numerous dark-red spots in the parietal pleura. **c** Pathological findings showed atypical spindle-like cells positive for ERG infiltrating the visceral pleura (arrows). **d** Chest X-ray showed expansion of the bilateral lung; however, pleurodesis was ineffective

A 71-year-old man with SA previously treated with chemoradiotherapy (8 courses of weekly Paclitaxel followed by radiation, and 9 courses of monthly Paclitaxel), was referred to our department for pneumothorax 10 months after initial diagnosis. He developed left pneumothorax, and air leakage was not controlled despite chest tube drainage. CT before developing pneumothorax showed multiple bullous lesions in the bilateral apex area (Fig. 2a). VATS was performed and the bullous lesion of the apex with air leakage was resected. The parietal pleura showed several dark-red spots, and we biopsied one of those (Fig. 2b). The pathological diagnosis was a metastasis of SA in the lung and parietal pleura. Pathologically, the tumor cells spread along with parietal and visceral pleura (Fig. 2c). There was no air leakage after surgery, and he was discharged on the fourth postoperative day. Seventeen days after the surgery, he was re-hospitalized for right pneumothorax and left hemopneumothorax. Chest tube drainage was done in both sides. After the full expansion of the bilateral lung (Fig. 2d), right pleurodesis was performed with Unitalc (4 g), and then with Minomycin (200 mg), and OK432 (5 KE), but the air leakage was not controlled. Left pleurodesis was done with Unitalc (4 g), but pleural effusion was not decreased. The patient died 43 days after the surgery due to deteriorating general condition.

## Discussion

SA is a rare malignancy in skin and considered aggressive of all cutaneous angiosarcomas. Predilection for lung metastasis is a feature of this disease, and 90% of all patients were reported to die from pulmonary complications, such as pneumothorax and hemothorax [1–3]. Pulmonary metastases of SA exhibit a variety of radiographic appearances, including thin wall cavities, sometimes looking like emphysematous bullae, which make diagnosis only by CT images challenging [4]. In these two patients, preoperative CT images showed thin wall cysts that mimicked emphysematous bullae. Since it is difficult to make a diagnosis only from CT, the pathological tissue diagnosis from the lung or parietal pleura is necessary in terms of clinical management. Generally, chemotherapy and molecular-targeted therapy (pazopanib) were considered as a treatment for metastatic SA; however, the development of pneumothorax and hemothorax made it difficult for patients to receive such a therapy.

In patient A, the development of empyema made it difficult to tell that the parietal pleura had metastatic SA. Apparently, cytology from pleural effusion was not enough for the diagnosis and histological examination of parietal pleura was necessary for diagnosis. Considering the possibility that metastasis in the parietal pleura had already existed in the first surgery, debridement might have caused uncontrolled hemothorax afterward in this patient. Consequently, the suspected uncontrolled infection made it impossible to give him chemotherapy.

In patient B, we obtained the pathological diagnosis of metastatic SA from the resected lung and biopsy of the parietal pleura. We recognized an impressive intrathoracic finding of dark-red spots in the parietal pleura which can be the characteristic lesion of the parietal pleura metastasis of SA before developing hemothorax. To our knowledge, this thoracoscopic finding is reported for the first time in the literature. The patient had hemothorax in the left side after surgery, which was not controlled by repeated pleurodesis despite full expansion of the lung. Deterioration of the general condition was very rapid after the recurrence of pneumothorax and; therefore, we missed a chance to treat with chemotherapy.

In general, pleurodesis using talc or OK-432 is considered effective in carcinomatous pleurisy when the lung is fully expanded by chest tube drainage [5]; however, it was not successful for pneumothorax and hemothorax in patient B. The mechanisms of pleural metastasis of SA are mentioned in the literature [1, 4]. Namely, (i) excavation of a solid nodular lesion, (ii) infiltration of tumor cells into the walls of preexisting bullous lung tissue, (iii) infiltration of malignant cells into the walls of air sacs and cystic distension through the ball-valve effect of the tumor, and (iv) tumor cell proliferation to form blood-filled cystic spaces that are characteristic of angiosarcoma. The pathological findings from these two patients suggest that the angiosarcomas that spread to the parietal pleura resulted in hemothorax, and the ones that spread to the visceral pleura caused thin wall cysts that consequently developed pneumothoraxes. The extensive infiltration of tumor cells along with the parietal and visceral pleura can make it difficult to manage pneumothorax and hemothorax with pleurodesis even if the lung is fully expanded. Notably, these infiltrating tumor cells did not make nodule or mass lesions, and it was difficult to predict from imaging.

## Conclusion

This report firstly describes metastatic SA lesions in the parietal pleura before causing hemothorax. In patients with a history of SA who develop pneumothorax and hemothorax, surgical biopsy rather than cytology is essential for diagnosis. Surgeons must recognize that conventional surgical intervention or pleurodesis will have unsatisfactory results.

## Data Availability

Data sharing is not applicable to this article, as no datasets were generated or analyzed during the study.

## Abbreviations

SA: Scalp angiosarcoma
VATS: Video-assisted thoracoscopic surgery
CT: Computed tomography
